# Risk Factors for Subacute Thyroiditis Recurrence: A Systematic Review and Meta-Analysis of Cohort Studies

**DOI:** 10.3389/fendo.2021.783439

**Published:** 2021-12-23

**Authors:** Jing Zhang, Guoyong Ding, Jingru Li, Xiao Li, Lin Ding, Xiangyun Li, Shuxiang Yang, Fang Tang

**Affiliations:** ^1^ Department of Endocrinology and Metabology, The First Affiliated Hospital of Shandong First Medical University and Shandong Provincial Qianfoshan Hospital, Jinan, China; ^2^ Center for Big Data Research in Health and Medicine, The First Affiliated Hospital of Shandong First Medical University and Shandong Provincial Qianfoshan Hospital, Jinan, China; ^3^ Organization and Personnel Section, Weifang Municipal Center for Disease Control and Prevention, Weifang, China; ^4^ School of Public Health, Shandong First Medical University & Shandong Academy of Medical Sciences, Taian, China; ^5^ Department of Quality Control, Anqiu City People's Hospital, Weifang, China; ^6^ Department of Clinical Pharmacy, The First Affiliated Hospital of Shandong First Medical University & Shandong Provincial Qianfoshan Hospital, Jinan, China; ^7^ Department of Clinical Pharmacy, Shandong Provincial Qianfoshan Hospital, Shandong University, Jinan, China; ^8^ School of Public Health, Weifang Medical University, Weifang, China; ^9^ Shandong Provincial Qianfoshan Hospital, Cheeloo College of Medicine, Shandong University, Jinan, China

**Keywords:** subacute thyroiditis, recurrence rate, risk factors, meta-analysis, cohort study

## Abstract

**Background:**

Subacute thyroiditis (SAT) is a self-limited inflammatory thyroid disease with recurring episodes. However, the results regarding the recurrence rate and risk factors for SAT are inconsistent. This meta-analysis aimed to summarize the evidence of the recurrence rate and the risk factors for SAT.

**Methods:**

The present study involved the performance of a systematic literature search of all English studies published in PubMed, Embase, Web of Science, and The Cochrane Library from inception to August 20, 2021. Cohort studies that reported the SAT recurrence rate and risk factors for recurrence were included. Two independent investigators extracted relevant information. Fixed- and random-effects models were used to pool effect sizes based on study heterogeneity.

**Results:**

Eighteen cohort studies were identified. The pooled findings showed that the recurrence rate was 12.0% (95% CI: 8.2%, 17.1%). The risk of recurrence in the glucocorticoids group was higher than that in the NSAIDs group (RR = 1.84, 95% CI: 1.04, 3.24). However, there was no significant difference in age or sex between the recurrence group and the non-recurrence group. Findings from one or two cohort studies also indicated that the copresence of HLA-B*18:01 and -B*35, the number of days required to taper prednisolone (PSL) to 5 mg/day, the duration of disease before treatment less than 30 days, the sialic acid level, or the TSH level at the termination of treatment and further extension of the hypoechoic area and increase in thyroid volume were related to the recurrence of SAT.

**Conclusion:**

Recurrence was common in SAT patients. The present study indicated that glucocorticoid treatment was associated with a higher recurrence rate of SAT than NSAIDs treatment. The clinical implications of this association should be interpreted with caution, and further clinical trials on the long-term effects of different treatment strategies are needed.

## Introduction

Subacute thyroiditis (SAT), also known as granulomatous thyroiditis, giant cell thyroiditis, and de Quervain thyroiditis, accounts for 5% of all clinical thyroid abnormalities (1, 2). The peak incidence occurs at 30 – 50 years, and women are affected three times more frequently than men (3, 4). It is generally believed that the occurrence of SAT is related to viral infection or autoimmune response, and susceptibility is related to human leukocyte antigen (HLA), mainly related to HLA-B*35, HLA-B*18:01, DRB1*01, and C*04:01 (3, 5–7).

SAT is a self-limited inflammatory thyroid disease, that usually has three phase course. The first phase of the acute inflammatory process destroys the thyroid follicles and releases thyroid hormones into the circulatory system, resulting in thyrotoxicosis. Then, the thyroid is depleted of stored thyroid hormone, and a phase of hypothyroidism typically occurs. Finally, thyroid hormone and thyroid-stimulating hormone (TSH) levels return to normal as the disease subsides, usually within 12 months (8, 9). However, some patients may experience recurrence or permanent hypothyroidism during follow-up (5, 10). The incidence rate of SAT has maintained an upward trend in recent years (11). Recurrence and prolonged treatment time have become severe problems for the treatment of SAT (12, 13).

Recurrence is usually defined as the relapse of episodes of pain with elevated laboratory parameters erythrocyte sedimentation rate (ESR) or C-reactive protein (CRP) and ultrasonographic findings (14). Many studies have reported recurrence rates, but depending on the studied population, discrepancies in the SAT recurrence rate vary significantly between studied groups, ranging from 0% to over 30% (15, 16). Recurrence occurs when the PSL dose is gradually reduced during treatment and even many years after the first attack (17, 18). SAT recurrence can seriously affect the lives of patients and create psychological troubles for them. Thus, the determination of the risk groups for recurrent SAT can guide clinicians in preventing early recurrence and provide early diagnosis and proper treatment. Although studies have investigated the risk factors for the recurrence of SAT in certain areas, endocrinologists’ knowledge gap on SAT relapse remains to be addressed. To our knowledge, no study has systematically and comprehensively reviewed the SAT recurrence rate and risk factors for SAT recurrence through meta-analysis. This study aimed to conduct a systematic review and meta-analysis of cohort studies to estimate SAT recurrence rates and summarize the risk factors for SAT recurrence.

## Materials and Methods

This study followed the Meta-Analysis of Observational Studies in Epidemiology (MOOSE) (**Supplementary Table 1**) and the Preferred Reporting Items for Systematic Review and Meta-Analysis (PRISMA) guidelines (**Supplementary Table 2**) (19, 20).

### Literature Search

We used a comprehensive search strategy to identify relevant English language literature in the following electronic databases: PubMed, Embase, Web of Science, and The Cochrane Library (up to August 20, 2021). The full search strategy is shown in **Supplementary Table 3**, and includes Medical Subject Headings (MeSH) headings and free term searches for “subacute thyroiditis”, “de Quervain thyroiditis”, “recurrence” and “cohort study”. The subjects of the studies were defined as humans, and the language of the articles was limited to English. We also manually searched reference lists from the included studies to identify potential additional eligible studies.

### Study Selection

The inclusion criteria were as follows: (1) cohort study; (2) patients diagnosed with SAT based on their clinical diagnosis (12, 21, 22); (3) baseline and follow-up number of patients ≥ 10; and (4) the study reported the SAT recurrence rates or odds ratios (ORs), relative risks (RRs), hazard ratios (HRs) with 95% confidence intervals (CIs) of risk factors, and equivalent data. If multiple articles were published from the same cohort, the most informative report was included. Articles that did not meet the eligibility criteria were excluded.

Two authors (JZ and JL) independently screened titles and abstracts initially, and full-text articles were evaluated to ensure that they met the eligible inclusion criteria. If there were disagreements that could not be resolved through discussion, another author (FT) was invited to make a decision.

### Data Extraction and Quality Assessment

Data were extracted from each of the included studies. The extracted data included the first author of the study, publication year, country, sample characteristics (e.g., sample size, mean age or range, the number of females), duration of follow-up, recurrence rate, treatment, risk factors investigated, significant risk factors and related effect size (e.g., ORs, RRs, HRs) with 95% CIs. Risk factors for recurrence included general characteristics (e.g., age, sex), therapy, HLA haplotype, laboratory parameters and ultrasonography.

The Newcastle-Ottawa Quality Scale (NOS) (23) was used to assess the quality of the included cohort studies. It consists of eight items and three components: selection, comparability and outcome. Additionally, the total stars range from 0 to 9. Studies with ≥ 7 stars were regarded as high-quality.

Data extraction and quality assessment were performed by two independent investigators (JZ and JL). Any disagreement was settled by discussion.

### Statistical Analysis

We performed a meta-analysis of the recurrence rate and risk factors associated with SAT recurrence. Heterogeneity between studies was assessed using Cochran’s Q statistic and *I^2^* values. *I^2^* described the percentage of total change due to heterogeneity between studies rather than chance. If the heterogeneity was high (*I^2^* > 50%), the random-effects model was adopted as the pooled method. Otherwise, the fixed-effects model was used. As the recurrence rate of SAT did not follow a normal distribution, logit transformation was adopted to transform the recurrence rate of SAT. For high heterogeneity, we used univariate meta-regression to explore the possible sources of between-study heterogeneity. Sensitivity analysis was used to assess the stability of the merger effect. A funnel plot and Egger’s test were used to estimate publication bias. Data were analyzed using R (R version 3.5.2; The R Foundation for Statistical Computing; Mathsoft, Cambridge, MA, USA). All tests were 2-sided, and *p* < 0.05 was considered statistically significant.

## Results

### Characteristics of Included Studies

**Figure 1:**
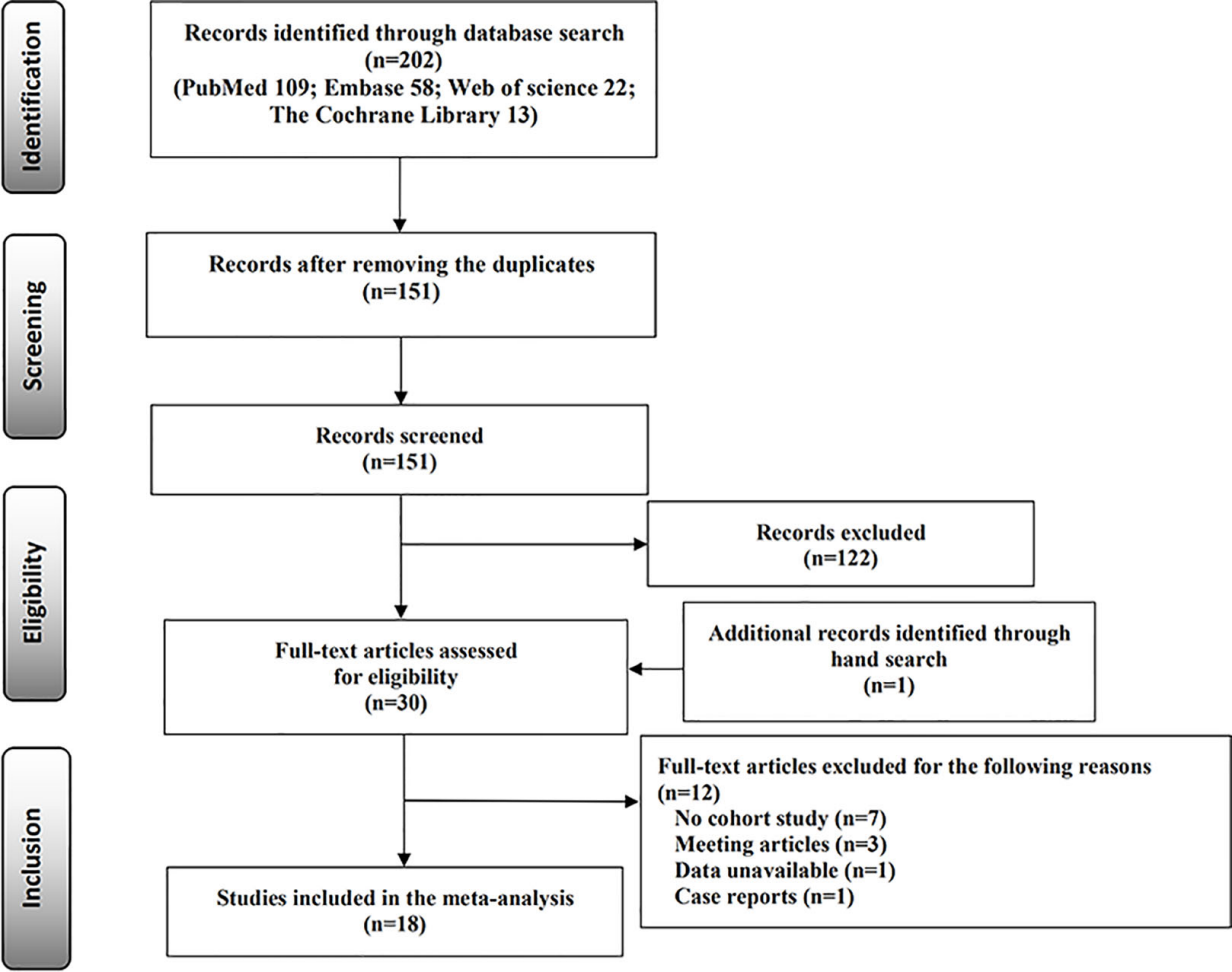
Flow diagram for the selection of the included studies.

A total of 202 published studies were identified through the electronic database search, including PubMed (n = 109), Embase (n = 58), Web of Science (n = 22), and The Cochrane Library (n = 13). After removing duplicate publications (n = 51) and reviewing the titles and abstracts, 122 studies were excluded. One study was identified through the reference lists of the existing relevant studies. We carefully read the full text and excluded 12 studies that did not meet the two criteria (**Supplementary Table 4**). Finally, 18 cohort studies were identified that met the inclusion criteria (11, 12, 14–18, 21, 22, 24–32). The selection process of the studies is displayed in the flow diagram (**Figure 1**).

**Tables 1**, **2** provide the characteristics, quality and main risk factors for all studies included in the review. The included studies were published between 1985 and 2021 among ten countries: Japan (n = 5) (17, 18, 22, 30, 31), Turkey (n = 4) (14, 24, 25, 28), Poland (n = 2) (12, 26), China (n = 1) (21), Denmark (n = 1) (16), Italy (n = 1) (32), Israel (n = 1) (27), the Kingdom of Saudi Arabia (n = 1) (15), Thailand (n = 1) (11), and the USA (n = 1) (29). The sample size of the included studies varied from 23 (15, 16) to 3,344 (18), and the duration of mean follow-up varied from 2 months (22) to 5 years (28). Sixteen studies (11, 12, 14–18, 21, 24–31) were of high quality and 2 studies (22, 32) were of moderate quality. The details of critical appraisal according to the NOS are presented in **Supplementary Table 5**.

**Table 1:** Characteristics and recurrence of included studies.

Author	Study year	Country	Study type	Total, Female	Mean age or age range(years)	Follow-up	No. of Recurrence	Recurrence rate (%)	NOS*
Hepsen et al., 2021 (24)	2017-2010	Turkey	Retrospective cohort	91, 72	NRG: 43 (28 - 71) RG: 39.5 (31 - 62)	3, 6, and 12 months	16	17.6	8
Sencar et al., 2020 (25)	2014-2019	Turkey	Retrospective cohort	247, 184	44 ± 7.5	29 months (range 6.2 - 70)	29	12	8
Li et al., 2019 (21)	2013-2016	China	Retrospective cohort	87, NR	Group1: 39.3 ± 5.7 Group2: 41.2 ± 4.6	4, 8 weeks, and 6 months	6	6.9	8
Sencar et al., 2019 (14)	2014-2018	Turkey	Retrospective cohort	217, 177	43 ± 9	27 months (range 6.2 - 64)	43	19.8	7
Stasiak et al., 2019 (12)	2003-2018	Poland	Retrospective cohort	49, 41	NRG: 44.4 RG: 42.7	NR	9	18.4	7
Stasiak et al., 2019 (26)	2003-2018	Poland	Retrospective cohort	64, 56	42.67 (27 - 69)	NR	9	14.1	7
Sato et al., 2017 (22)	2008-2014	Japan	Retrospective cohort	42, 33	48.8 ± 12.8	1st to 2nd: PSL 15.5 ± 4.1, NSAIDs 15.3 ± 9.0 days 2nd to 3rd: PSL 24.2 ± 9.3, NSAIDs 21.3 ± 7.8 days 3rd to 4th: PSL 28.2 ± 8.6, NSAIDs: 33.1 ± 10.2 days	4	9.5	6
Arao et al., 2015 (17)	2004-2013	Japan	Retrospective cohort	26, 23	49.0 ± 11.3 31.0 - 76.0	NR	4	15.4	7
Yotsapon et al., 2015 (11)	2007-2013	Thailand	Retrospective cohort	115, 102	43.8 ± 10.8	NR	14	12.2	7
Benbassat et al., 2007 (27)	1999-2005	Israel	Retrospective cohort	56, 39	48.6 ± 12	12 months	5	8.9	7
Erdem et al., 2007 (28)	1987-2001	Turkey	Retrospective cohort	169, 134	34.0 ± 17.8	5 years	21	12.4	8
Qari et al., 2005 (15)	2002-2004	Kingdom of Saudi Arabia	Prospective cohort	23, 15	35.8 ± 9.2 21 - 54	2 years	0	0	7
Fatourechi et al., 2003 (29)	1960-1997	USA	Retrospective cohort	94, 73	46 14 - 87	28 years	13	13.8	7
Mizukoshi et al., 2001 (30)	1997-1998	Japan	Retrospective cohort	36, 32	31 - 71	2 years	8	22.2	7
Bennedbaek et al., 1997 (16)	1993-1996	Denmark	Retrospective cohort	23, 17	43 32 - 68	18 months (range 6 - 33)	8	34.8	8
Iitaka et al., 1996 (18)	1970-1993	Japan	Retrospective cohort	3,344, 3,032	1st: 38.4 ± 6.3 2nd: 53.1 ± 8.9 3rd: 57.8 ± 10.1 Range:14 - 75	NR	48	1.4	8
Tajiri et al., 1993 (31)	NR	Japan	Prospective cohort	43, 38	27 - 68	NR	14	32.6	7
Madeddu et al., 1985 (32)	NR	Italy	Prospective cohort	Total: 38, 30 Follow-up:12	17 - 68	2 - 4 months	1	8.3	6

NOS, Newcastle-Ottawa Scale; NR, not reported; NRG, non-recurrence group; NSAIDs, nonsteroidal anti-inflammatory drugs; PSL, prednisolone; RG, recurrence group.

*The quality of the studies assessed using the Newcastle-Ottawa Scale.

**Table 2:** Risk factors of included studies.

Author	Risk factors investigated	Significant risk factors
Hepsen et al., 2021 (24)	General characteristics: age, sex	NS. age, *p* = 0.24; sex, *p* = 0.51.
	Parameters at the time of diagnosis: ESR, CRP, TSH, FT4, FT3, aTPO, aTg Parameters at the end of the first treatment: ESR, CRP, TSH, FT4, cumulative MPS dose, total treatment duration	At the time of diagnosis: NS. ESR, *p* = 0.89; CRP, *p* = 0.88; TSH, *p* = 0.07; FT4, *p* = 0.07; FT3, *p* = 0.76; aTPO, *p* = 0.63; aTg, *p* = 0.24. At the end of the first treatment: NS. ESR, *p* = 0.95; CRP, *p* = 0.56; TSH (mIU/L), NRG 3.1 (0.2 - 37), RG 1.1 (0.01 - 6.7), *p* < 0.0001; FT4 (ng/dL), NRG 0.8 (0.48 - 1.73), RG 0.9 (0.65 - 1.48), *p* = 0.019; cumulative MPS dose (mg), NRG 500 (420 - 924), RG 1424 (840 - 2268), *p* < 0.0001; total treatment duration (d), NRG 42 (42 - 52), RG 84 (42 - 126), *p* < 0.0001.
Sencar et al., 2020 (25)	General characteristics: age, sex	NS. age, *p* = 0.24; sex, *p* = 0.12.
	Laboratory parameters: total leukocytes, neutrophils, ESR, CRP, TSH, FT4, FT3, aTPO, aTg	NS. total leukocytes, *p* = 0.26; neutrophils, *p* = 0.38; ESR, *p* = 0.95; CRP, *p* = 0.89; TSH, *p* = 0.09; FT4, *p* = 0.08; FT3, *p* = 0.56; aTPO, *p* = 0.56; aTg, *p* = 0.61.
	Ultrasonography	NS. bilateral/unilateral disease, *p* = 0.39; thyroid volume, *p* = 0.65.
Li et al., 2019 (21)	Treatment (PSL + PV/PSL)	NS, *p* = 0.350.
Sencar et al., 2019 (14)	Treatment(methylprednisolone/NSAIDs)	Treatment: Patients treated with only NSAIDs had 6 recurrence, patients treated with only steroid had 21 recurrence, *p* = 0.040.
Stasiak et al., 2019 (12)	General characteristics: age, sex	NS. mean age, *p* = 0.675; sex, *p* = 1.000.
	HLA haplotype	HLA haplotype: HLA-B*18:01 + B*35 ± C*04:01 (n), NRG 2 RG 4, *p* = 0.007.
	Laboratory parameters: TSH, FT4, FT3, aTPO, aTg, TRAb, ESR, CRP, WBC, 25-hydroxy vitamin D	Laboratory parameters (M ± SD). TSH (mIU/L), NRG 0.101 ± 0.232, RG 0.646 ± 0.773, *p = *0.015; FT4 (ng/dL), NRG 3.029 ± 1.604, RG 1.79 ± 0.628, *p* = 0.006; FT3 (pg/mL), NRG 7.237 ± 3.88, RG 4.332 ± 1.189, *p* = 0.025; aTPO (IU/mL), NRG 36.54 ± 63.3, RG 12.773 ± 3.349, *p* = 0.029. NS. aTg, *p* = 0.394; TRAb, *p* = 0.726; ESR, *p* = 0.381; CRP, *p* = 0.156; WBC, *p* = 0.322; 25-hydroxy vitamin D, *p* = 0.736.
Stasiak et al., 2019 (26)	NA	Recurrence may be related to studied population (Caucasian and Asian).
Sato et al., 2017 (22)	Treatment (PSL/NSAIDs)	NS, *p* = 0.635.
Arao et al., 2015 (17)	General characteristics: age, sex, clinical score	NS. age, *p* = 0.851; sex, *p* = 0.790; clinical score, *p* = 0.817.
	Laboratory parameters: FT4, CRP, ESR, Leukocyte count, Tg	NS. FT4, *p* = 0.512; CRP, *p* = 0.626; ESR, *p* = 0.703; Leukocyte count, *p* = 0.871; Tg, *p* = 0.685.
	Treatment: first dose of PSL, total treatment time, total dose of PSL and presence or absence of creeping thyroiditis, the initial dose of PSL and the number of days required to taper PSL to 5 mg/day	The number of days required to taper PSL to 5 mg/day: NRG 44.3 ± 15.3, RG: 19.0 ± 11.9, *p* = 0.012.
Yotsapon et al., 2015 (11)	NR	NR
Benbassat et al., 2007 (27)	NR	NR
Erdem et al., 2007 (28)	General characteristics: age, sex	NS. age, *p* = 0.337; sex, *p* = 0.840.
	Treatment (PSL/NSAIDs)	NS. treatment, *p* = 0.317.
	ultrasonography	NS. ultrasonography, *p* = 0.232.
	Laboratory parameters: aTg, aTPO	NS
Qari et al., 2005 (15)	NR	NR
Fatourechi et al., 2003 (29)	NR	NR
Mizukoshi et al., 2001 (30)	General characteristics: sex	NS. sex, *p* = 0.180.
	Laboratory parameters (M ± SD): ESR, WBC, CRP, SA, Tg, FT3, FT4	NS. ESR (mm/h), NRG 86.5 ± 28.1, RG 74.5 ± 35.6; WBC (×10^2^/mm^3^), NRG 63.7 ± 15.9 RG 67.8 ± 13.4; CRP (mg/dL), NRG 3.0 ± 3.4 RG 3.3 ± 2.5; SA (mg/dL), NRG 101.4 ± 16.4 RG 102.0 ± 20.5; Tg (pmol/L), NRG 1,242.0 ± 1,915.9 RG 980.9 ± 882.5; FT3 (pmol/L), NRG 11.03 ± 4.42 RG 11.06 ± 4.16; FT4 (pmol/L), NRG 44.03 ± 25.17 RG 36.68 ± 16.26.
	Treatment: duration of PSL treatment (10 mg/day)	Duration of PSL treatment (10 mg/day) may be the risk factor.
Bennedbaek et al., 1997 (16)	Ultrasonography	The further extension of hypoechoic areas and increase in thyroid volume were associated with recurrence.
Iitaka et al., 1996 (18)	General characteristics: age	NS. age in first episode (year): 38.4 ± 6.3; second episode: 53.1 ± 8.9; third episode: 57.8 ± 10.1.
	Laboratory parameters: ESR, Latent period, T3, T4, FT3, FT4, RAIU	ESR (M ± SD, 2nd vs. 1st, mm/h), 57 ± 26, *p* < 0.02; RAIU (M ± SD, 3rd vs. 1st, %), 6.0 ± 6.0, *p* < 0.05.
	Treatment: treatment (PSL/NSAIDs/none), duration of treatment in different recurrence episode	Duration of treatment (M ± SD, 2nd vs 1st, month): 1.9 ± 0.9, *p* < 0.002.
Tajiri et al., 1993 (31)	General characteristics: age, sex	NS. age, *p* = 0.230; sex, *p* = 0.390.
	Laboratory parameters: ESR, T3, T4, CRP, SA levels at termination of treatment	SA levels at termination of treatment: *p* < 0.01.
	Treatment: the duration of disease before treatment	Duration of disease before treatment less than 30 days: NRG 24.2 ± 15.2 RG 14.1 ± 9.2, *p* < 0.05.
Madeddu et al., 1985 (32)	Laboratory parameters: Tg, thyrotropin (basal and after stimulation with protirelin), T3, T4, FT3, and FT4 radioactive iodine uptake, scanning, and ESR	Tg level was accompanied by a rise in free T3 and T4 levels above normal and by a fall in thyrotropin values.

aTg, anti-thyroglobulin antibodies; aTPO, anti-thyroid peroxidase antibodies; CRP, C-reactive protein; ESR, erythrocyte sedimentation rate; FT3, free triiodothyronine; FT4, free thyroxine; M, mean; MPS, methylprednisolone; NA, not applicable; NGR, non-recurrence group; NR, not reported; NSAIDs, nonsteroidal anti-inflammatory drug; NS, not statistically significant; PSL, prednisolone; PV, Prunella vulgaris; RAIU, radioactive iodine uptake test; RG, recurrence group; RR, relative risk; SA, sialic acid; SD, standard deviation; T3, triiodothyronine; T4: serum thyroxine; Tg, thyroglobulin; TRAb, thyrotropin receptor antibodies; TSH, thyroid-stimulating hormone; WBC, white blood count.

### Recurrence Rate

**Figure 2:**
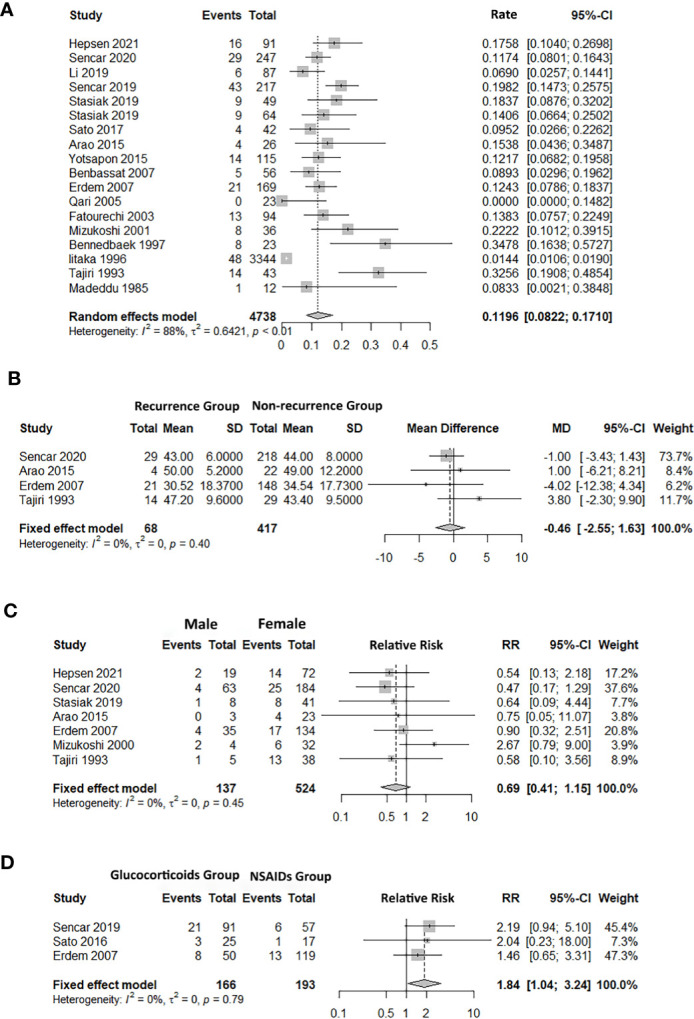
Forest plot of SAT recurrence rate and the association between factors and subacute thyroiditis (SAT) recurrence. **(A)** recurrence rate. **(B)** age. **(C)** sex. **(D)** treatment (glucocorticoids/NSAIDs). 95% CI, confidence interval.

A total of 18 studies reported the recurrence rate of SAT. The recurrence rates varied from 0% (15) to 34.8% (16). The pooled recurrence rate was 12.0% (95% CI: 8.2%, 17.1%), with significant heterogeneity (*I^2^* = 87.9%, *p* < 0.01) (**Figure 2A**). The results of univariate meta-regression indicated that publication year (coefficient = 0.14, *p* = 0.80), sample (coefficient = -0.53, *p* = 0.21), country (coefficient = 0.52, *p* = 0.27), follow-up period (coefficient = 0.05, *p *= 0.86), and study type (coefficient = 0.03, *p* = 0.96) were not sources of heterogeneity (**Supplementary Table 6**).

### Risk Factors for the SAT Recurrence

#### General Characteristics

Five studies reported the age difference for recurrence (17, 18, 25, 28, 31). The pooled finding suggested that there was no significant difference in age between the recurrence group (RG) and the non-recurrence group (NRG) (Age MD = -0.46, 95% CI: -2.55, 1.63; **Figure 2B**).

Seven studies referred to the recurrence and non-recurrence situation of males and females, and the number of recurrence of males was less than that of females (12, 17, 24, 25, 28, 30, 31). However, the pooled recurrence rate showed that there was no significant difference in sex between the RG and NRG groups (RR = 0.69, 95% CI: 0.41, 1.15; **Figure 2C**).

#### Therapy

Hepsen et al. compared low- and high-dose steroids in the treatment of SAT, and the findings showed that high-dose steroids had a higher SAT recurrence rate than low-dose steroids (24). Five studies focused on the effects of different treatments (14, 18, 21, 22, 28), and three of them reported the recurrence outcome between glucocorticoids and NSAIDs (14, 22, 28). The pooled result showed that the risk of recurrence in the glucocorticoids group was higher than the NSAIDs group (RR = 1.84, 95% CI: 1.04, 3.24; **Figure 2D**). The number of days required to taper the PSL dose to 5 mg/day (NRG: 44.3 ± 15.3, RG: 19.0 ± 11.9, *p* = 0.012) and the duration of the disease before therapy less than 30 days were also associated with the recurrence rate (17, 31).

#### HLA Haplotype

Stasiak et al. explored the relationship between the HLA haplotype and recurrence (12). The findings showed that the risk of SAT recurrence depended on HLA, and the determining factor was the copresence of HLA-B*18:01 and -B*35.

#### Laboratory Parameters, Ultrasonography

Hepsen et al. reported that the TSH level at the end of the treatment was a predictor of recurrence (24). Stasiak et al. found that TSH, free triiodothyronine (FT3), and free thyroxine (FT4) were significantly different between RG and NRG and that elevated anti-thyroid peroxidase antibody (aTPO) concentration at the first SAT episode was a protective factor (**Table 2**) (12). Moreover, sialic acid levels at the termination of treatment were an important risk factor (31). In addition, Bennedbaek et al. indicated that further extension of the hypoechoic area and an increase in thyroid volume were risk factors for SAT recurrence (16).

#### Sensitivity Analysis and Publication Bias

**Figure 3:**
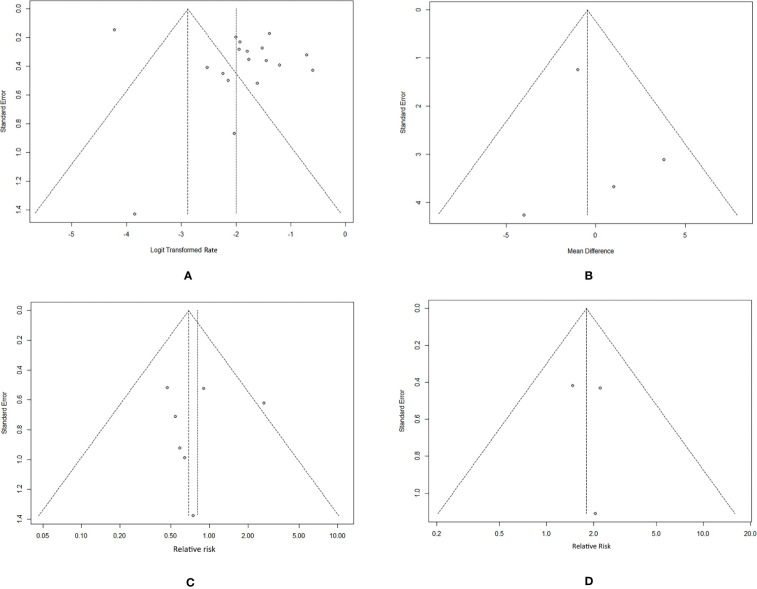
Funnel plots of the SAT recurrence rate and risk factors. **(A)** recurrence rate. **(B)** age. **(C)** sex. **(D)** treatment (glucocorticoids/NSAIDs).

In the sensitivity analysis, no individual study substantially influenced the pooled recurrence rate of SAT (**Supplementary Figure 1**). The funnel plot can visually assess publication bias, and the horizontal line represents summary effect estimates. The points on the funnel plot of the recurrence rate did not fall onto the line (**Figure 3A**). Egger’s test of recurrence rate indicated no publication bias (*p* = 0.1137). The funnel plots and Egger’s tests of factors indicated no statistically significant potential for publication bias in the assessment of recurrence risk factors: age (*p* = 0.7066), sex (*p* = 0.8735), and treatment (*p* = 0.8632) (**Figures 3B–D**).

## Discussion

To the best of our knowledge, this is the first meta-analysis examining the recurrence rates and risk factors for SAT more comprehensively using data from cohort studies. Our study confirmed that 12.0% of SAT patients experienced recurrence. Previous studies reported that the occurrence of SAT is common in middle-aged women, but the current results of this study indicated that age and sex are not statistically significantly associated with SAT recurrence (27, 33).

For SAT therapy, these included studies mainly reported two different treatments, glucocorticoids (PSL and methylprednisolone)/NSAIDs and PSL + PV (*Prunella vulgaris*)/PSL. The current main purpose of SAT treatment is to relieve symptoms. The American Thyroid Association recommends corticosteroids to treat severe patients, but it does not provide objective criteria defining severe cases. The standard recommendation is to use prednisone 40 mg/day for 1 - 2 weeks, and then gradually taper the dose (34). Medication is generally based on clinical experience. As all included studies were retrospective cohort studies, and there was no significant difference in clinical background between the glucocorticoids group and the NSAIDs group, we cannot find the basis for treatment options (14, 22, 28). In terms of symptom relief, the view that corticosteroid treatment is superior to NSAIDs has been widely accepted, especially considering the aspect of the quicker effect, faster pain relief, etc. (22, 29, 35). Nevertheless, side effects and SAT recurrence are prone to occur in association with corticosteroid treatment (11, 16, 29, 30). The pooled findings of this study also suggested that the risk of recurrence caused among glucocorticoid-treated group is higher than that in the NSAIDs-treated group (RR = 1.84, 95% CI: 1.04, 3.24). This finding is consistent with those of previous studies and the underlying cause of recurrence may be that glucocorticoids are used to treat severe patients or patients who do not respond to NSAIDs. These patients are more likely to have a recurrence, and premature discontinuation of glucocorticoids can also lead to recurrence (35–37). Hepsen et al. showed that the recurrence rate of high-dose steroids is higher than that of low-dose steroids, which may be related to high-dose steroids that may promote virus replication and are more likely to cause recurrence (24). Kubota et al. believed that 15 mg/day PSL can be applied to Japanese people (38). Koirala et al. treated SAT with an initial dose of 20 mg/day and observed no adverse effects (39). Although these studies were not randomized controlled trials, they still suggested that low-dose PSL may reduce the recurrence rate and have an excellent therapeutic effect. Similarly, Soltani et al. reviewed latest studies regarding the most appropriate dosage of prednisolone with the lowest recurrence rate in the treatment of patients with subacute granulomatous thyroiditis, suggest that 15 - 20 mg/day of prednisolone is the best choice (40).

In addition, the duration of PSL administration is a potential risk factor. During PSL treatment, when tapering PSL from 10 mg/day to 5 mg/day, SAT recurrence is most likely to occur. Meanwhile, if the PSL is stopped too early, the pain may be more likely to recur. Therefore, at least six weeks for a therapeutic period before tapering PSL to 5 mg/day is highly suggested by clinicians to prevent recurrences, and extending the duration of PSL treatment at 10 mg/day may decrease the recurrence rate (17, 30). In addition, Tajiri et al. indicated that the time to be treated is a crucial factor for recurrence. In particular, patients who initiated PSL treatment within 30 days of onset had a higher recurrence rate than those who experienced a longer duration of illness prior to the treatment because inflammation of the thyroid gland may be improved in the natural course of the disease (31). In the future, we need to pay attention to the treatment dose and treatment duration to obtain more evidence that affects the high recurrence rate of PSL treatment. We cannot draw conclusions about the prognosis of PSL combined with PV in SAT treatment because only one included study explored the recurrence of PSL + PV/PSL treatment, and there was no significant difference between the two groups. This study suggested that we can focus on combination drugs in the future to enhance the therapeutic effect and reduce the recurrence rate.

Yamamoto et al. reported recurrence after ten years in three cases and suggested that *HLA-A26* may be related to the predisposition to SAT recurrence (41). However, this was a case report and no other studies have proven that *HLA-A26* is a risk factor for recurrence thus far. Stasiak et al. confirmed that the copresence of HLA-B*18:01 and -B*35 is the decisive factor of recurrence through a high resolution HLA haplotype (12). Patients with this HLA haplotype are more likely to experience recurrence, and high-risk patients with recurrence can be screened by identifying HLA haplotypes. The latest case report reported that three siblings with SAT lacked the copresence of HLA-B*18:01 and -B*35, but one of them had three episodes of recurrence, which may be related to the existence of some other HLA alleles. The coexistence of these HLA alleles may increase or decrease the susceptibility to recurrence (42). These results indicated that the impact of the HLA genotype on recurrence is complex and important. We need research on the potential relationship between more HLA haplotypes and recurrence to support these conclusions.

Hepsen et al. suggested that the TSH level at the end of treatment was associated with recurrence (24). Stasiak et al. found that TSH, FT3, and FT4 were significantly different between RG and NRG (12). Moreover, the increase in aTPO concentration during the first episode of SAT is a protective factor (12). In addition, Tajiri et al. suggested that the sialic acid level at the termination of treatment is a risk factor (31). Due to the inconsistent measurement time of laboratory parameters, we cannot obtain more reliable evidence. Three included studies focused on the ultrasonography of SAT recurrence (16, 25, 28). The finding of Bennedbaek et al. showed that recurrence is related to the further extension of hypoechoic areas and an increase in thyroid volume and has nothing to do with the extension of hypoechogenicity or initial thyroid function (16). Both Sencar et al. and Bennedbeak et al. mentioned that the initial thyroid volume is not related to recurrence (16, 25). Similarly, there was no difference in the type of nodules shown by ultrasound between the RG and NRG (28).

Recurrences of SAT may occur soon after the initial therapy, but they also happen even many years after the first attack (41, 43). In the included studies, Iitaka et al. also reported that 48 out of 3,344 SAT patients (1.4%) had multiple recurrences over 24 years (18). The data showed that symptoms during recurrence were generally milder than those in the first episode and the incubation period seemed to shorten as patients aged. The faster response of the immune system may make the symptoms of recurrent reactions milder (18).

This meta-analysis has several strengths. First, we conducted a comprehensive literature search, and the included studies were cohort studies that could provide more convincing results than case-control studies, cross-sectional studies or sporadic case reports. Second, the sample size was large, with a total of 4,764 SAT patients, and the follow-up time was long, with an average of 4 years. Third, accurate estimation of the recurrence rate and comprehensive research on risk factors can enable clinicians to provide better consultations for patients who have experienced the first SAT. Fourth, it compared the two main SAT treatments in the clinic, which can provide clinical guidance for clinicians. However, the potential limitations of this meta-analysis should be considered. First, most of the included studies were retrospective cohort studies, and the quality of historical clinical data may not be guaranteed. Second, because SAT is a rare type of thyroiditis, most of the included studies reported fewer than 100 patients and limited information on the risk factors for SAT recurrence. These limitations may impose a modest constraint on interpreting these findings, but they should not substantively undermine the internal validity of our study. Third, our main purpose was to explore the recurrence rate and the risk factors for SAT, therefore, we did not pay attention to the long-term prognosis of the disease.

In summary, our study demonstrated that 12.0% of patients might develop SAT recurrence. Moreover, the risk of recurrence in patients treated with glucocorticoids is higher than of patients treated with of NSAIDs. Treatment-related factors, HLA haplotype, the sialic acid level, or the TSH level at the termination of treatment, further extension of the hypoechoic area and increase in thyroid volume were all potential predictors for recurrence of SAT. Further randomized controlled trials, prospective cohort studies, and studies on the molecular and cellular mechanisms are needed to explore the association between these factors and SAT recurrence. Moreover, the choice of treatment should also consider the impact on the long-term prognosis of patients, such as thyroid function. It is recommended to carry out more clinical studies of different therapies to observe the prognosis and long-term effects of SAT patients. Our findings might shed light on the choice of therapeutic optimization for clinicians to reduce recurrence and have important implications for improving the quality of life of SAT patients in the future.

## Data Availability Statement

The original contributions presented in the study are included in the article/**Supplementary Material**. Further inquiries can be directed to the corresponding author.

## Author Contributions

Study concept and design: FT. Acquisition, analysis, interpretation of data: JZ, GD, XiaoL, JL, and LD. Drafting of the manuscript: JZ. Critical revision of the manuscript for important intellectual content: FT, GD, and LD. Statistical analysis: JZ, GD, XianL, and JL. Administrative, technical, or material support: All authors. All authors have approved the final draft of the manuscript.

## Funding

This study was supported by grants from National Natural Science Foundation of China (71804093), Academic Promotion Programme of Shandong First Medical University (2019LJ005), Project of Priority Research from Department of Science and Technology of Shandong Province (2020RKB14114), and Science and Technology development Plan of Traditional Chinese Medicine in Shandong Province (2019-0374).

## Conflict of Interest

The authors declare that the research was conducted in the absence of any commercial or financial relationships that could be construed as a potential conflict of interest.

## Publisher’s Note

All claims expressed in this article are solely those of the authors and do not necessarily represent those of their affiliated organizations, or those of the publisher, the editors and the reviewers. Any product that may be evaluated in this article, or claim that may be made by its manufacturer, is not guaranteed or endorsed by the publisher.
