# Supplementary material for: Risk Factors for Subacute Thyroiditis Recurrence: A Systematic Review and Meta-Analysis of Cohort Studies
Source: Front Endocrinol (Lausanne). 2021 Dec 23;12:783439. doi: 10.3389/fendo.2021.783439 (PMC8734029; doi:10.3389/fendo.2021.783439)
Supplement: Supplementary file 1 [file DataSheet_1.docx]

Supplementary Material

# Supplementary Tables and figure

## Supplementary Tables

**Supplementary Table 1.** MOOSE checklist.

| **Item No** | **Recommendation** | **Reported**  **on Page No** |
| --- | --- | --- |
| **Reporting of background should include** | | |
| 1 | Problem definition | 2 |
| 2 | Hypothesis statement | 2 |
| 3 | Description of study outcome(s) | 2 |
| 4 | Type of exposure or intervention used | 2 |
| 5 | Type of study designs used | 2 |
| 6 | Study population | 2 |
| **Reporting of search strategy should include** | | |
| 7 | Qualifications of searchers (eg, librarians and investigators) | 2 |
| 8 | Search strategy, including time period included in the synthesis and key words | 2 |
| 9 | Effort to include all available studies, including contact with authors | 2 |
| 10 | Databases and registries searched | 2 |
| 11 | Search software used, name and version, including special features used (eg, explosion) | 2 |
| 12 | Use of hand searching (eg, reference lists of obtained articles) | 2 |
| 13 | List of citations located and those excluded, including justification | 2 |
| 14 | Method of addressing articles published in languages other than English | 2 |
| 15 | Method of handling abstracts and unpublished studies | NA |
| 16 | Description of any contact with authors | 2 |
| **Reporting of methods should include** | | |
| 17 | Description of relevance or appropriateness of studies assembled for assessing the  hypothesis to be tested | 3 |
| 18 | Rationale for the selection and coding of data (eg, sound clinical principles or convenience) | 3 |
| 19 | Documentation of how data were classified and coded (eg, multiple raters, blinding and interrater reliability) | 3 |
| 20 | Assessment of confounding (eg, comparability of cases and controls in studies where  appropriate) | NA |
| 21 | Assessment of study quality, including blinding of quality assessors, stratification or regression on possible predictors of study results | 3 |
| 22 | Assessment of heterogeneity | 3 |
| 23 | Description of statistical methods (eg, complete description of fixed or random effects models, justification of whether the chosen models account for predictors of study results, dose-response models, or cumulative meta-analysis) in sufficient detail to be replicated | 3 |
| 24 | Provision of appropriate tables and graphics | NA |
| **Reporting of results should include** | | |
| 25 | Graphic summarizing individual study estimates and overall estimate | 4 - 8 |
| 26 | Table giving descriptive information for each study included | 5 - 7 |
| 27 | Results of sensitivity testing (eg, subgroup analysis) | 4 |
| 28 | Indication of statistical uncertainty of findings | NA |
| **Reporting of discussion should include** | | |
| 29 | Quantitative assessment of bias (eg, publication bias) | 4 |
| 30 | Justification for exclusion (eg, exclusion of non-English language citations) | 3 |
| 31 | Assessment of quality of included studies | 3 |
| **Reporting of conclusions should include** | | |
| 32 | Consideration of alternative explanations for observed results | 4 - 5, 8 - 9 |
| 33 | Generalization of the conclusions (ie, appropriate for the data presented and within the domain of the literature review) | 9 |
| 34 | Guidelines for future research | 9 |

Abbreviations: NA, not applicable.

**Supplementary Table 2.** PRISMA checklist.

| **Section/topic** | **#** | **Checklist item** | **Reported on page #** |
| --- | --- | --- | --- |
| **TITLE** |  |  |  |
| Title | 1 | Identify the report as a systematic review, meta-analysis, or both. | 1 |
| **ABSTRACT** |  |  |  |
| Structured summary | 2 | Provide a structured summary including, as applicable: background; objectives; data sources; study eligibility criteria, participants, and interventions; study appraisal and synthesis methods; results; limitations; conclusions and implications of key findings; systematic review registration number. | 1 - 2 |
| **INTRODUCTION** |  |  |  |
| Rationale | 3 | Describe the rationale for the review in the context of what is already known. | 2 |
| Objectives | 4 | Provide an explicit statement of questions being addressed with reference to participants, interventions, comparisons, outcomes, and study design (PICOS). | 2 |
| **METHODS** |  |  |  |
| Protocol and registration | 5 | Indicate if a review protocol exists, if and where it can be accessed (e.g., Web address), and, if available, provide registration information including registration number. | NA |
| Eligibility criteria | 6 | Specify study characteristics (e.g., PICOS, length of follow-up) and report characteristics (e.g., years considered, language, publication status) used as criteria for eligibility, giving rationale. | 2 |
| Information sources | 7 | Describe all information sources (e.g., databases with dates of coverage, contact with study authors to identify additional studies) in the search and date last searched. | 2 |
| Search | 8 | Present full electronic search strategy for at least one database, including any limits used, such that it could be repeated. | 2 |
| Study selection | 9 | State the process for selecting studies (i.e., screening, eligibility, included in systematic review, and, if applicable, included in the meta-analysis). | 2 |
| Data collection process | 10 | Describe method of data extraction from reports (e.g., piloted forms, independently, in duplicate) and any processes for obtaining and confirming data from investigators. | 2 |
| Data items | 11 | List and define all variables for which data were sought (e.g., PICOS, funding sources) and any assumptions and simplifications made. | 2 |
| Risk of bias in individual studies | 12 | Describe methods used for assessing risk of bias of individual studies (including specification of whether this was done at the study or outcome level), and how this information is to be used in any data synthesis. | NA |
| Summary measures | 13 | State the principal summary measures (e.g., risk ratio, difference in means). | 2 - 3 |
| Synthesis of results | 14 | Describe the methods of handling data and combining results of studies, if done, including measures of consistency (e.g., *I^2^*) for each meta-analysis. | 3 |
| Risk of bias across studies | 15 | Specify any assessment of risk of bias that may affect the cumulative evidence (e.g., publication bias, selective reporting within studies). | 3 |
| Additional analyses | 16 | Describe methods of additional analyses (e.g., sensitivity or subgroup analyses, meta-regression), if done, indicating which were pre-specified. | 3 |
| **RESULTS** |  |  |  |
| Study selection | 17 | Give numbers of studies screened, assessed for eligibility, and included in the review, with reasons for exclusions at each stage, ideally with a flow diagram. | 3 |
| Study characteristics | 18 | For each study, present characteristics for which data were extracted (e.g., study size, PICOS, follow-up period) and provide the citations. | 5 |
| Risk of bias within studies | 19 | Present data on risk of bias of each study and, if available, any outcome level assessment (see item 12). | NA |
| Results of individual studies | 20 | For all outcomes considered (benefits or harms), present, for each study: (a) simple summary data for each intervention group (b) effect estimates and confidence intervals, ideally with a forest plot. | 3 - 4, 7 |
| Synthesis of results | 21 | Present results of each meta-analysis done, including confidence intervals and measures of consistency. | 3 - 4, 7 |
| Risk of bias across studies | 22 | Present results of any assessment of risk of bias across studies (see Item 15). | 4 |
| Additional analysis | 23 | Give results of additional analyses, if done (e.g., sensitivity or subgroup analyses, meta-regression [see Item 16]). | 3 - 4 |
| **DISCUSSION** |  |  |  |
| Summary of evidence | 24 | Summarize the main findings including the strength of evidence for each main outcome; consider their relevance to key groups (e.g., healthcare providers, users, and policy makers). | 4 - 5, 8 - 9 |
| Limitations | 25 | Discuss limitations at study and outcome level (e.g., risk of bias), and at review-level (e.g., incomplete retrieval of identified research, reporting bias). | 9 |
| Conclusions | 26 | Provide a general interpretation of the results in the context of other evidence, and implications for future research. | 9 |
| **FUNDING** |  |  |  |
| Funding | 27 | Describe sources of funding for the systematic review and other support (e.g., supply of data); role of funders for the systematic review. | 9 |

Abbreviations: NA, not applicable.

**Supplementary Table 3.** Actual search strategies.

***PubMed Search Strategy***

#1. (Subacute Thyroiditides.ti,ab.) OR (Subacute Thyroiditis,ti,ab.)OR (Thyroiditides, Subacute.ti,ab) OR (Subacute Painful Thyroiditis.ti,ab) OR( Painful Thyroiditides, Subacute.ti,ab) OR (Painful Thyroiditis, Subacute.ti,ab) OR (Subacute Painful Thyroiditides.ti,ab) OR Thyroiditides, Subacute Painful.ti,ab) OR (Thyroiditis, Subacute Painful.ti,ab) OR (Granulomatous Thyroiditis.ti,ab) OR (Granulomatous Thyroiditides.ti,ab) OR (Thyroiditides, Granulomatous.ti,ab) OR (Thyroiditis, Granulomatous.ti,ab) OR (Subacute Nonsuppurative Thyroiditis.ti,ab) OR (Nonsuppurative Thyroiditides, Subacute.ti,ab) OR (Nonsuppurative Thyroiditis, Subacute.ti,ab) OR (Subacute Nonsuppurative Thyroiditides.ti,ab) OR (Thyroiditides, Subacute Nonsuppurative.ti,ab) OR (Thyroiditis, Subacute Nonsuppurative.ti,ab) OR (De Quervain Thyroiditis.ti,ab) OR (Thyroiditis, De Quervain.ti,ab) OR (Giant Cell Thyroiditis.ti,ab) OR (Cell Thyroiditides, Giant.ti,ab) OR (Cell Thyroiditis, Giant.ti,ab) OR (Subacute lymphocytic thyroiditis.ti,ab)OR (Subacute granulomatous thyroiditis.ti,ab) OR (Palpation thyroiditis.ti,ab) OR (Subacute Postpartum thyroiditis.ti,ab) OR (thyroiditis, giant cell.ti,ab) OR (giant cell thyroiditides.ti,ab) OR (thyroiditides, giant cell.ti,ab) OR ("Thyroiditis, Subacute"[Mesh])

#2. ("Recurrence"[Mesh]) OR ("Retreatment"[Mesh]) OR (recur*.ti,ab) OR (recrudescence*.ti,ab) OR (relapse*.ti,ab) OR (retreatment*.ti,ab)

#3. ("Cohort Studies"[Mesh]) OR ("Follow-Up Studies"[Mesh]) OR (cohort*) OR (followed) OR (followed up) OR (retrospective*) OR (observe*)

#4 #1 AND #2 AND #3

#5 limit #4 to English language

***Embase Search Strategy***

#1. ('subacute thyroiditis'/exp) OR ('subacute thyroiditis':ab,ti )OR ('subacute thyroiditides':ab,ti )OR ('thyroiditides, subacute':ab,ti) OR ('subacute painful thyroiditis':ab,ti) OR ('painful thyroiditides, subacute':ab,ti) OR ('painful thyroiditis, subacute':ab,ti) OR ('subacute painful thyroiditides':ab,ti) OR ('thyroiditides, subacute painful':ab,ti) OR ('thyroiditis, subacute painful':ab,ti) OR ('granulomatous thyroiditis':ab,ti) OR ('granulomatous thyroiditides':ab,ti) OR ('thyroiditides, granulomatous':ab,ti) OR ('thyroiditis, granulomatous':ab,ti) OR ('subacute nonsuppurative thyroiditis':ab,ti) OR ('nonsuppurative thyroiditides, subacute':ab,ti) OR ('nonsuppurative thyroiditis, subacute':ab,ti) OR ('subacute nonsuppurative thyroiditides':ab,ti) OR ('thyroiditides, subacute nonsuppurative':ab,ti) OR ('thyroiditis, subacute nonsuppurative':ab,ti) OR ('de quervain thyroiditis':ab,ti) OR ('thyroiditis, de quervain':ab,ti) OR ('giant cell thyroiditis':ab,ti) OR ('cell thyroiditides, giant':ab,ti) OR ('cell thyroiditis, giant':ab,ti) OR ('giant cell thyroiditides':ab,ti) OR ('thyroiditides, giant cell':ab,ti) OR ('thyroiditis, giant cell':ab,ti) OR ('subacute granulomatous thyroiditis':ab,ti) OR ('subacute lymphocytic thyroiditis':ab,ti) OR ('subacute postpartum thyroiditis':ab,ti OR ('palpation thyroiditis':ab,ti)

#2. ('recurrent disease'/exp) OR ('retreatment'/exp） OR (recur*:ab,ti ）OR (retreatment*:ab,ti) OR (recrudescence*:ab,ti) OR (relapse*:ti,ab)

#3. ('cohort analysis'/exp) OR ('follow up'/exp) OR (cohort*) OR (followed) OR ('followed up') OR (retrospective*) OR (observe*)

#4 #1 AND #2 AND #3

#5 limit #4 to (English language and embase)

***Web of science Search Strategy***

#1. TS=(Subacute Thyroiditides OR Subacute Thyroiditis OR Thyroiditides, Subacute OR Subacute Painful Thyroiditis OR Painful Thyroiditides, Subacute OR PainfulThyroiditis, Subacute OR Subacute Painful Thyroiditides OR Thyroiditides, Subacute Painful OR Thyroiditis, SubacutePainful OR Granulomatous Thyroiditis OR Granulomatous Thyroiditides OR Thyroiditides, Granulomatous OR Thyroiditis, Granulomatous OR Subacute Nonsuppurative Thyroiditis OR Nonsuppurative Thyroiditides, Subacute OR NonsuppurativeThyroiditis, Subacute OR Subacute Nonsuppurative Thyroiditides OR Thyroiditides, Subacute Nonsuppurative OR Thyroiditis, SubacuteNonsuppurative OR De Quervain Thyroiditis OR Thyroiditis, De Quervain OR Giant Cell Thyroiditis OR Cell Thyroiditides, Giant OR Cell Thyroiditis, Giant OR Giant Cell Thyroiditides OR Thyroiditides, Giant Cell OR Thyroiditis, Giant Cell OR Subacute granulomatous thyroiditis OR Subacute lymphocytic thyroiditis OR Subacute Postpartum thyroiditis OR Palpation thyroiditis)

#2 TS=(retreatment* OR relapse* OR recrudescence* OR recur*)

#3 TS=(chort* OR chort study OR follow-up OR followed OR “followed up”OR retrospective* OR observe*)

#4 #1 AND #2 AND #3

#5 limit #4 to English language

***The Cochrane Library Search Strategy***

#1 MeSH descriptor: [Thyroiditis, Subacute] explode all trees

#2 (Subacute Thyroiditides OR Subacute Thyroiditis OR Thyroiditides, Subacute OR Subacute Painful Thyroiditis OR Painful Thyroiditides, Subacute OR Painful Thyroiditis, Subacute OR Subacute Painful Thyroiditides OR Thyroiditides, Subacute Painful OR Thyroiditis, Subacute Painful OR Granulomatous Thyroiditis OR Granulomatous Thyroiditides OR Thyroiditides, Granulomatous OR Thyroiditis, Granulomatous OR Subacute Nonsuppurative Thyroiditis OR Nonsuppurative Thyroiditides, Subacute OR Nonsuppurative Thyroiditis, Subacute OR Subacute Nonsuppurative Thyroiditides OR Thyroiditides, Subacute Nonsuppurative OR Thyroiditis, Subacute Nonsuppurative OR De Quervain Thyroiditis OR Thyroiditis, De Quervain OR Giant Cell Thyroiditis OR Cell Thyroiditides, Giant OR Cell Thyroiditis, Giant OR Giant Cell Thyroiditides OR Thyroiditides, Giant Cell OR Thyroiditis, Giant Cell OR Subacute granulomatous thyroiditis OR Subacute lymphocytic thyroiditis OR Subacute Postpartum thyroiditis OR Palpation thyroiditis):ti,ab,kw

#3 (retreatment* OR relapse* OR recrudescence* OR recur*):ti,ab,kw

#4 MeSH descriptor: [Recurrence] explode all trees

#5 MeSH descriptor: [Retreatment] explode all trees

#6 #3 OR #4 OR #5

#7 #1 OR #2

#8 MeSH descriptor: [Cohort Studies] explode all trees

#9 MeSH descriptor: [Follow-Up Studies ] explode all trees

#10 (cohort* OR followed OR “followed up” OR retrospective* OR observe*)

#11 #8 OR #9 OR #10

#12 #6 AND #7 AND #11

#13 limit #12 to English language

**Supplementary Table 4.** List of included and excluded studies

| Included and excluded reasons | No. studies (n) | References |
| --- | --- | --- |
| Included studies | 18 | (1) (2) (3) (4) (5) (6) (7) (8) (9) (10) (11) (12) (13) (14) (15) (16) (17) (18) |
| No cohort study | 7 | (19) (20) (21) (22) (23) (24) (25) |
| Meeting articles | 3 | (26) (27) (28) |
| Data unavailable | 1 | (29) |
| Case reports | 1 | (30) |

**REFERENCES**

1. Hepsen S, Akhanli P, Sencar ME, Duger H, Sakiz D, Kizilgul M, et al. The Evaluation of Low- and High-Dose Steroid Treatments in Subacute Thyroiditis: A Retrospective Observational Study. *Endocr Pract* (2020) 27:594-600. doi: 10.1016/j.eprac.2020.11.009.

2. Sencar ME, Calapkulu M, Sakiz D, Akhanli P, Hepsen S, Duger H, et al. The contribution of ultrasonographic findings to the prognosis of subacute thyroiditis. *Arch Endocrinol Metab* (2020) 64:306-311. doi: 10.1016/j.eprac.2020.11.009.

3. Li F, Wu Y, Chen L, Hu L, Liu X. Initial treatment combined with Prunella vulgaris reduced prednisolone consumption for patients with subacute thyroiditis. *Ann Transl Med* (2019) 7:45. doi: 10.21037/atm.2019.01.07.

4. Sencar ME, Calapkulu M, Sakiz D, Hepsen S, Kus A, Akhanli P, et al. An Evaluation of the Results of the Steroid and Non-steroidal Anti-inflammatory Drug Treatments in Subacute Thyroiditis in relation to Persistent Hypothyroidism and Recurrence. *Sci Rep* (2019) 9:16899. doi: 10.1038/s41598-019-53475-w.

5. Stasiak M, Tymoniuk B, Stasiak B, Lewinski A. The Risk of Recurrence of Subacute Thyroiditis Is HLA-Dependent. *Int J Mol Sci* (2019) 20:1089. doi: 10.3390/ijms20051089.

6. Stasiak M, Michalak R, Stasiak B, Lewinski A. Clinical characteristics of subacute thyroiditis is different than it used to be - current state based on 15 years own material. *Neuro Endocrinol Lett* (2019) 39:489-495.

7. Sato J, Uchida T, Komiya K, Goto H, Takeno K, Suzuki R, et al. Comparison of the therapeutic effects of prednisolone and nonsteroidal anti-inflammatory drugs in patients with subacute thyroiditis. *Endocrine* (2017) 55:209-214. doi: 10.1007/s12020-016-1122-3.

8. Arao T, Okada Y, Torimoto K, Kurozumi A, Narisawa M, Yamamoto S, et al. Prednisolone Dosing Regimen for Treatment of Subacute Thyroiditis. *J UOEH* (2015) 37:103-110. doi: 10.7888/juoeh.37.103.

9. Yotsapon T, Sirinate K, Siriwan B, Soontaree N, Thep H. Clinical Features and Outcomes of Subacute Thyroiditis in Thai Patients. *Journal of the ASEAN Federation of Endocrine Societies* (2015) 30:125-128. doi: 10.1210/jc.2002-021799.

10. Benbassat CA, Olchovsky D, Tsvetov G, Shimon I. Subacute thyroiditis: clinical characteristics and treatment outcome in fifty-six consecutive patients diagnosed between 1999 and 2005. *J Endocrinol Invest* (2007) 30:631-635. doi: 10.1007/BF03347442.

11. Erdem N, Erdogan M, Ozbek M, Karadeniz M, Cetinkalp S, Ozgen AG, et al. Demographic and clinical features of patients with subacute thyroiditis: results of 169 patients from a single university center in Turkey. *J Endocrinol Invest* (2007) 30:546-550. doi: 10.1007/BF03346347.

12. Qari FA, Maimani AA. Subacute thyroiditis in Western Saudi Arabia. *Saudi Med J* (2005) 26:630-633.

13. Fatourechi V, Aniszewski JP, Fatourechi GZ, Atkinson EJ, Jacobsen SJ. Clinical features and outcome of subacute thyroiditis in an incidence cohort: Olmsted County, Minnesota, study. *J Clin Endocrinol Metab* (2003) 88:2100-2105. doi: 10.1210/jc.2002-021799.

14. Mizukoshi T, Noguchi S, Murakami T, Futata T, Yamashita H. Evaluation of recurrence in 36 subacute thyroiditis patients managed with prednisolone. *Intern Med* (2001)40:292-295. doi: 10.2169/internalmedicine.40.292.

15. Bennedbaek FN, Hegedüs L. The Value of Ultrasonography in the Diagnosis and Follow-up of Subacute Thyroiditis. *Thyroid* (1997) 7:45-50. doi: 10.1089/thy.1997.7.45.

16. Iitaka M, Momotani N, Ishii JKI. Incidence of Subacute Thyroiditis Recurrences after a Prolonged Latency: 24-Year Survey. *J Clin Endocrinol Metab* (1996) 81:466-469. doi: 10.1210/jcem.81.2.8636251.

17. Tajiri J, Noguchi S, Morita M, Tamaru M, Murakami T, Murakami N. Serum sialic acid levels in the diagnosis and follow-up of subacute granulomatous thyroiditis. *Endocr J* (1993) 40:83-87. doi: 10.1507/endocrj.40.83.

18. Madeddu G, Casu AR, Costanza C, Marras G, Arrasl ML, Marrosu A, et al. Serum thyroglobulin levels in the diagnosis and follow-up of subacute 'painful' thyroiditis. A sequential study. *Arch Intern Med* (1985)145:243-247.

19. Tang C, Dong Y, Lu L, Zhang N. C-reactive protein and thyroid-stimulating hormone levels as risk factors for hypothyroidism in patients with subacute thyroiditis. *Endocr Connect* (2021)10:965-972. doi: 10.1530/EC-21-0212.

20. Calapkulu M, Sencar ME, Sakiz D, Duger H, Ozturk Unsal I, Ozbek M, et al. The prognostic and diagnostic use of hematological parameters in subacute thyroiditis patients. *Endocrine* (2020) 68:138-143. doi: 10.1007/s12020-019-02163-w.

21. Calapkulu M, Sencar ME, Sakiz D, Unsal IO, Ozbek M, Cakal E The Importance of Vitamin D Level in Subacute Thyroiditis Disease and the Effect of Vitamin D on Disease Prognosis. *Endocr Pract* (2020) 26:1062-1069. doi: 10.4158/EP-2020-0046.

22. Hernik A, Szczepanek-Parulska E, Filipowicz D, Czarnywojtek A, Wrotkowska E, Kramer L, et al. Hepcidin and Iron Homeostasis in Patients with Subacute Thyroiditis and Healthy Subjects. *Mediators Inflamm* (2019) 2019:5764061doi: 10.1155/2019/5764061.

23. Lian D. Short-term Prednisone to Treat STA Study(SPTSS) (2013). https://clinicaltrialsgov/show/NCT01837433. [Accessed March 15, 2021].

24. Martinez DS, Chopra IJ. Use of oral cholecystography agents in the treatment of hyperthyroidism of subacute thyroiditis. *Panminerva Med* (2003) 45:53-57.

25. Yamamoto M, Saito S, Kaise K, Kaise N, Yoshida K, Yoshinaga K. Changes in thyroid hormones by treatment with aspirin and prednisolone in subacute thyroiditis with hyperthyroidism. *Tohoku J Exp Med* (1979) 127:85-95. doi: 10.1620/tjem.127.85.

26. Lian D. An efficiency and safety of short-term prednisone treating to moderate and severe subacute thyroiditis. Endocrine reviews Conference: 96th annual meeting and expo of the endocrine society, ENDO 2014 Chicago, IL united states Conference start: 20140621 Conference end: 20140624 Conference publication: (varpagings) 35.

27. Yokozawa T, Shimada T. Is percutaneous methylpredonisolone acetate (MA) injection a new option for the treatment of patients with prolonged subacute thyroiditis? *Thyroid* (2009) 19:S37.

28. Radian N 1973 Phenylbutazone in the treatment of subacute thyroiditis. *REVROUMENDOCR* (1973) 10:471-475.

29. Duan L, Zheng H. Short-course prednisone treatment for moderate to severe subacute thyroiditis-a prospective, randomized, controlled, single-blind study (SPTSS study, clinicaltrials id: nct01837433). *Thyroid* (2016) 26:A51.

30. Yamamoto M, Saito S, Sakurada T, Tamura M, Kudo T, Yoshida K, et al. Recurrence of subacute thyroiditis over 10 years after the first attack in three cases. *Endocrinol Jpn* (1988) 35:833-839. doi: 10.1507/endocrj1954.35.833.

**Supplementary Table 5.** The NOS stars of included studies

| Author | Selection | Comparability | Outcome | Total stars (out of 9) |
| --- | --- | --- | --- | --- |
| Hepsen et al.,2020 | 4 | 1 | 3 | 8 |
| Sencar et al.,2020 | 4 | 1 | 3 | 8 |
| Li et al.,2019 | 4 | 1 | 3 | 8 |
| Sencar et al.,2019 | 4 | 1 | 2 | 7 |
| Stasiak et al.,2019 | 4 | 1 | 2 | 7 |
| Stasiak et al.,2019 | 4 | 1 | 2 | 7 |
| Sato et al.,2017 | 4 | 1 | 1 | 6 |
| Arao et al.,2015 | 4 | 1 | 2 | 7 |
| Yotsapon et al.,2015 | 4 | 1 | 2 | 7 |
| Benbassat et al.,2007 | 4 | 1 | 2 | 7 |
| Erdem et al.,2007 | 4 | 1 | 3 | 8 |
| Qari et al.,2005 | 4 | 0 | 3 | 7 |
| Fatourechi et al.,2003 | 4 | 1 | 2 | 7 |
| Mizukoshi et al.,2001 | 4 | 1 | 2 | 7 |
| Bennedbaek et al.,1997 | 4 | 1 | 3 | 8 |
| Iitaka et al.,1996 | 4 | 1 | 3 | 8 |
| Tajiri et al.,1993 | 4 | 1 | 2 | 7 |
| Madeddu et al.,1985 | 4 | 1 | 1 | 6 |

**Supplementary Table 6.** Univariate meta-regression of study characteristics

| Variables | Coefficient, 95% CI | SE | *p*-value |
| --- | --- | --- | --- |
| Publication year | 0.14 (-0.89, 1.16) | 0.52 | 0.80 |
| Sample | -0.53 (-1.37, 0.31) | 0.43 | 0.21 |
| Country | 0.52 (-0.40, 1.44) | 0.47 | 0.27 |
| Follow-up period | 0.05 (-0.50, 0.60) | 0.28 | 0.86 |
| Study type | 0.03 (-1.28, 1.35) | 0.67 | 0.96 |

Abbreviations: CI, confidence interval.

## Supplementary Figure


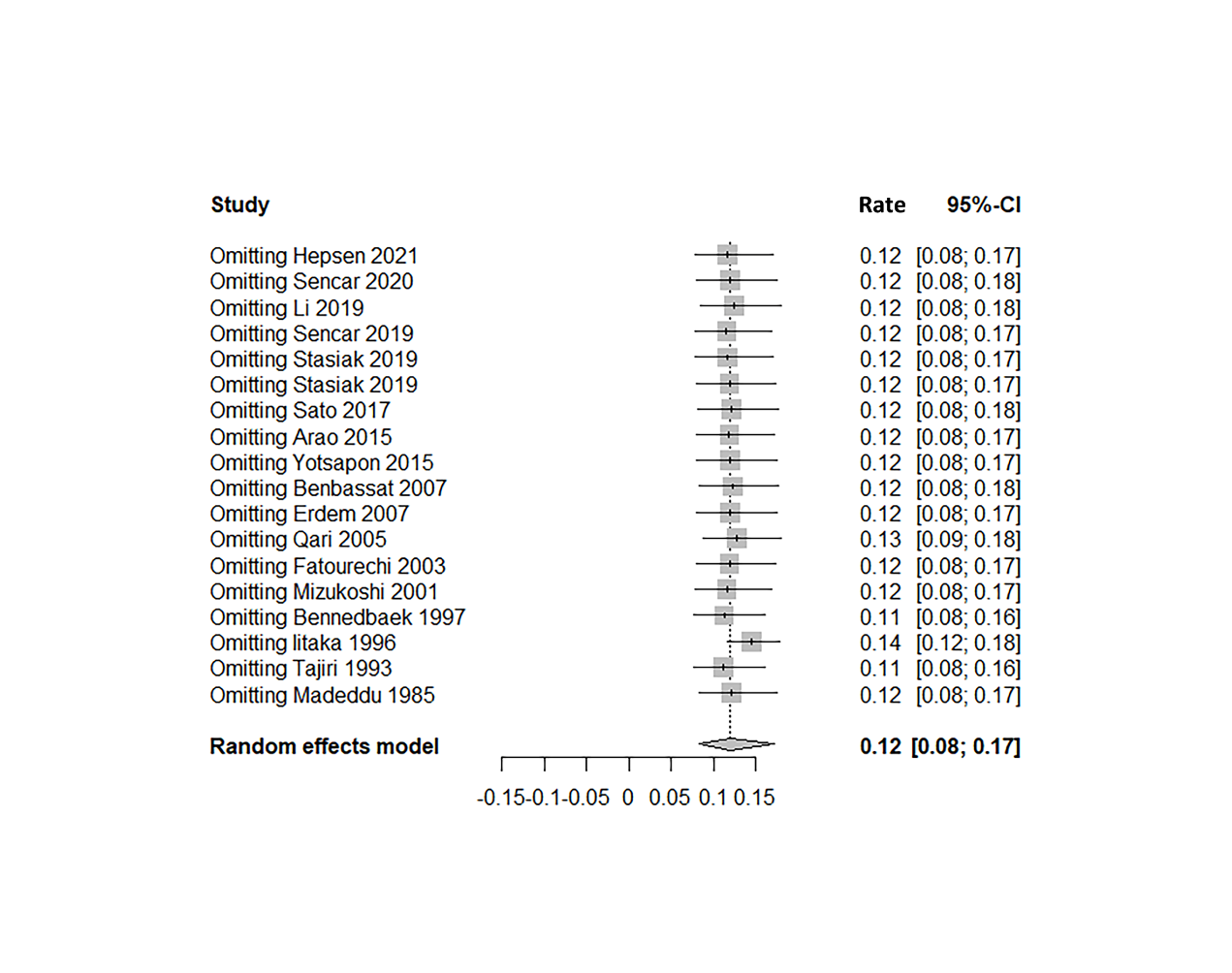


**Supplementary Figure 1.** Sensitivity analysis of SAT recurrence rate
